# Spatial structure and network characteristics of the coupling coordination innovation ecosystems in the Guangdong–Hong Kong–Macao Greater Bay area

**DOI:** 10.1038/s41598-023-50771-4

**Published:** 2024-01-03

**Authors:** Zhichen Yang, Xiangtao Li, Fangfang Wang, Rongjian Chen, Renwen Ma

**Affiliations:** 1 School of Economics, Jinan University, Guangzhou, 510632 China; 2 School of Digital Economics, Guangdong University of Finance & Economics, Foshan, 528100 China; 3 School of Science and Engineering, The Chinese University of Hong Kong, Shenzhen, 518172 China

**Keywords:** Statistics, Environmental economics, Socioeconomic scenarios, Sustainability

## Abstract

In recent times, a new wave of scientific and technological advancements has significantly reshaped the global economic structure. This shift has redefined the role of regional innovation, particularly in its contribution to developing the Guangdong–Hong Kong–Macao Greater Bay area (GBA) into a renowned center for science, technology, and innovation. This study constructs a comprehensive evaluation system for the Regional Innovation Ecosystem (RIE). By applying the coupling coordination degree model and social network analysis, we have extensively analyzed the spatial structure and network attributes of the coupled and coordinated innovation ecosystem in the GBA from 2010 to 2019. Our findings reveal several key developments: (1) There has been a noticeable rightward shift in the kernel density curve, indicating an ongoing optimization of the overall coupling coordination level. Notably, the center of gravity for coupling coordination has progressively moved southeast. This shift has led to a reduction in the elliptical area each year, while the trend surface consistently shows a convex orientation toward the center. The most significant development is observed along the ‘Guangdong–Shenzhen–Hong Kong–Macao Science and Technology Innovation Corridor’, where the level of coupling coordination has become increasingly pronounced. (2) The spatial linkages within the GBA have been strengthening. There are significant spatial transaction costs in the regional innovation ecological network. In the context of the 2019 US-China trade war, the cities of Jiangmen and Zhaoqing experienced a notable decrease in connectivity with other cities, raising concerns about their potential marginalization. (3) Guangzhou, Shenzhen, and Hong Kong have emerged as core nodes within the network. The network exhibits a distinctive “core–edge” spatial structure, characterized by both robustness and vulnerability in various aspects.

## Introduction

Since the theory of the national innovation system emerged in China’s academic circles in the 1990s, the issue of innovation system construction has become another research focus of Chinese scholars studying regional innovation. In contrast to the innovation environment of other city clusters in China, GBA faces the unique situation of “two social systems, three legal systems, and three independent customs territories”. It makes the institutional conflict of cross-regional collaborative governance in the process of promoting the coordinated development of regional innovation in the GBA prominent. At the same time, GBA is both an important space for China to participate in international cooperation and competition, and a model for realizing high-level coordinated economic development first. Therefore, studying the spatial structure and network characteristics of the coupled and coordinated RIE in GBA can meet the two-way demand for the implementation of national strategies and the coordinated development of the regional economy.

China is in a critical phase of accelerating its transformation into an innovative nation. It is essential to accurately recognize the significant shift in the innovation paradigm and understand the Innovation Ecosystem (IE) theory. This understanding is crucial for improving the quality of regional collaborative innovation across the country. This open system encourages interactive learning both within the system and between the system and the environment. The innovation paradigm has formally entered the stage of a RIE supported by the theory of evolutionary economics, along with the development trend of a diverse and composite global economy^1^. Starting from different perspectives such as symbiosis theory, synergetics, and systematics, scholars have used symbiotic evolution models, principal component analysis, panel data regression, and fuzzy set qualitative comparative analysis to evaluate the construction^2,3^, characterization^4,5^, coordination evaluation^6–8^, and evolutionary trends^9–11^ for research. Among them, Katz explored the problems of commonly used performance indicators in complex innovation systems in Canada, proposed the concept of size adjustment, and demonstrated its potential advantages more accurately. It assessed the contribution of groups of different sizes to the innovation system by examining size-independence indicators^3^; Yan et al. used a systems-thinking approach to study the effects of Taiwan’s science parks and innovation policies on the IE and assess the innovation value of science parks^6^; Tsai and Chang and Möller and Halinen explore the prioritization of the influencing factors of regional innovation systems and the impact of each factor on the innovative network^3,4^; Shaw and Allen incorporate ecology to emphasize the existence of interconnected business modeling pathways in the network of IE in the United Kingdom. They validated these pathways with case studies to transfer material, information resources and value^5^; Carayannis et al. used a quadruple/quintuple helix innovation system model to dissect the enablers and implementers in the regional cooperation IE network and explore its coordinated development^7^; Li and Zhang used a symbiosis measurement model to calculate the degree of symbiosis of China’s RIE and analyze its impact on regional science and technology innovation^8^; Ranga et al. analyzed the role of the evolution of Japan’s University-Industry Collaboration (UIC) policy in shaping Japan’s IE^10^; Holgersson et al. analyzed the evolutionary trends of strategic IP management and IE using mobile communication systems as a case study. Then they emphasize the importance of shaping applicability regimes when formulating strategies in dynamic innovative environments^11^; Xu et al. constructed a spatial network matrix through gravitational modeling by using data on the number of joint patents in the Yangtze River Economic Belt. Then they analyzed its spatial linkages^12^. There are still four limits to the studies mentioned above. First, multi-dimensional analysis of the internal spatial structure of the IE coupling and coordination is generally lacking in research on RIE coupling and coordination. They are generally restricted to the measurement of indicators and assessment of the current situation. Second, studies on the coordination development of IE primarily concentrate on the global, national, or provincial levels. While studies on regions with higher levels of science and technology innovation, such as GBA, Beijing–Tianjin–Hebei region, and Yangtze River Delta economic belt, are only marginally insufficient to reflect the regional innovation characteristics in China. Thirdly, IE has a sizable network and complementary effects, just like natural ecosystems do. Some researchers have applied the concept of composite networks in ecology to the theoretical study of RIE^8,13–16^. However, the majority of the empirical evaluations that are currently available^17^ concentrate on the interactions of system members at the micro level. Studies on the structure and association characteristics of regional networks at the medium and macro levels are almost nonexistent. The majority of available empirical evaluations have concentrated on the interaction of system members at the micro level. Yet there is hardly any research on the characteristics of regional network structure and association at the medium and macro levels. However, social network analysis’s connection, balance, and dynamism are crucial for closing the regional innovation development gap and fostering regional innovation. Fourth, currently, most scholars pay more attention to the study of the structure of urban economic networks^18^. There are relatively few studies on regional innovation networks, with many scholars using cases to dissect RIE^5,11^. The construction of regional innovation networks is often limited to the use of co-authored paper data or joint patent data. Although some studies have tried to analyze the level of regional collaborative innovation by combining the spatial status of knowledge innovation networks and technological innovation networks^19–22^, it is still difficult to fully reflect the multidimensional evolutionary symbiosis network characteristics of RIE.

Building on the identified strengths and limitations of previous research, our study embarks on an innovative approach. We aim to merge ecosystem theory with complex network concepts to establish a Regional Innovation Network Ecosystem and its evaluation system. This ecosystem is characterized by the integrated coordination of two key elements: innovation liveliness and innovation habitat. Then Utilizing mathematical models and spatial statistics, our research investigates the interplay of coupling coordination between innovation live-liness and innovation habitat within the Guangdong–Hong Kong–Macao Greater Bay area (GBA). Finally, we delve into the spatial distribution structure of this coupling coordination within the Regional Innovation Ecosystem (RIE), employing the gravity model. Our primary contributions are as follows:

- A comprehensive RIE evaluation system based on the system level, guideline level and indicator level is constructed under the overall framework of GBA. The system comprehensively portrays the systematic characteristics of innovation activity and innovation habitats.
- We utilize the GBA as our research object to examine the spatial organization and coupling patterns of the RIE. This analysis provides support for the advancement of high-quality science and technology collaborative innovation within the GBA and may provide developmental insights for other innovative regions in China.
- Based on measuring the overall spatial structure of the coupled development of the RIE, we apply complex network theory to discern the distinct status and critical roles of each city within the innovation network. We investigate the multi-dimensional spatial structure and the intricate patterns of coupling coordination within the RIE. Exploring in detail the spatial connection mode of the coupling coordination among cities to reveal the “black box” system of regional innovation.

The remainder of the paper is structured as follows. The theoretical framework is presented in Part II. Part III introduces the materials and methods. Part IV provides empirical results. The conclusions and discussions are presented in Part V.

## Materials and methods

### Connotation of RIE

As the evolutionary laws of synergistic symbiosis and interactive adaptation in ecosystems are also suitable for innovation systems, studying regional innovation from an ecological perspective has gradually become a new research paradigm. The leap from a regional innovation system to an RIE highlights the characteristics of the dynamic, habitat, and growth of the innovation system^22^. Additionally, it is a renewal of the research perspective and research method. RIE refers to the system formed by the interdependence and interaction between innovation communities and the environment through the free circulation and organic aggregation of innovative materials and energy in a certain regional scope. Its concept emphasizes the interconnection of innovation subjects, factor resources, and auxiliary forces to form a dynamic innovation network, which further strengthens the stability of the system structure of regional innovation. In order to better characterize regional innovation and stay close to the actual context, we further introduce the ecological niche theory to explain the evolution of relationships within the RIE. Ecological niche theory reflects the reality and development potential of the subject through the attributes of “state” and “potential” dimensions. It can better reflect the relative position of a basic unit in the overall ecosystem in time and space, as well as its influence on the relationship between the unit and other units and functional utility.

**Figure 1:**
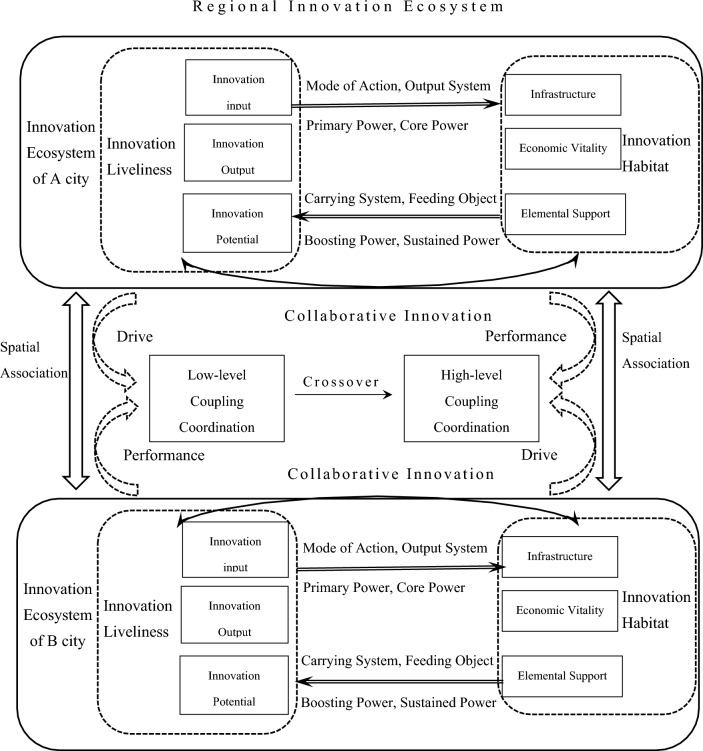
RIE coupling mechanism map.

We believe that the connotation of the RIE should be based on the dual definition of innovation liveliness and innovation habitat. Innovation liveliness represents the development status and vitality of the RIE, which is expressed by its attribute indicators such as innovation potential, innovation input, and innovation output. Innovation habitat represents the future survival and development trend of the RIE, which is composed of environmental factors such as infrastructure, economic vitality, and factor support that affect the development of innovation subjects. Innovation liveliness directly or indirectly participates in the whole process of science and technology innovation activities. It promotes technological innovation, and industrial upgrading, and optimizes the efficiency of innovation resource allocation by expanding the boundary of innovation potential, increasing the quantity of innovation input, and improving the quality of innovation output. This contributes to improving the overall spatial ecological quality of the innovative habitat. Innovation habitat permeates all aspects of innovation liveliness and provides the soil for innovation liveliness to nurture innovation achievements. Suitable innovation habitat conditions are conducive to accelerating the flow of innovation factors and resources. It can effectively reduce the external costs of innovation activities carried out by innovation subjects, give rise to diversified innovation demands, and promote the agglomeration and cooperation of regional innovation subjects. Thus promoting the efficient operation of innovation liveliness, improving the efficiency of scientific research and innovation, and the conversion rate of innovation achievements^23^. In the process of coupling coordination of innovation liveliness and innovation habitat, a continuous and stable innovation flow and feedback loop will be generated between the systems. The system will be continuously upgraded through continuous improvement of its structure, forming a well-adapted, mutual promotion and systematic and orderly regional innovation ecological model. In ecology, there are diverse connections of material, information and energy flow among various organisms within a community, forming a network system of compound cycles. Similarly, the nodes of a RIE also have a network structure. As can be seen from Fig. 1, innovation liveliness and innovation habitat provide the necessary guarantee for the normalized and self-organized operation of each innovation part through interactive coordination and energy conduction. The RIE is composed of the urban IE, and the organization and coordination of the RIE include the collaborative innovation relationship of innovation liveliness and innovation habitat within cities. Additionally, It also the spatial association relationship between the urban IE. Based on geographical proximity, spatial correlations generate knowledge spillover effects through exchanges and cooperation among innovation agents in different cities, exploiting the comparative advantages of “industry, academia, and research” in different cities to exert economies of scale. Moreover, it improves resource allocation efficiency through the interconnection of innovation factors in the region, thus positively influencing the overall regional innovation performance. This has a positive impact on the overall regional innovation performance. The regional innovation network is a highly visible expression of the spatial interrelationship among cities. In addition, it is the main form and carrier of the spatial and temporal relationships, organizational order, and system functions of the RIE.

### Construction of the index system of the RIE

When researchers create a regional innovation indicator system for GBA, they frequently choose only a small number of indicators to represent the overall system status or choose only nine cities in the Pearl River Delta as a research sample. They utilise it to represent the overall region, making the system less accurate when compared to other regions in China^24,25^. Based on the above analysis, the evaluation system structure based on system level, criterion level and indicator level was refined considering the availability and consistency of the existing research results^26,27^ and the data of GBA. We had an attempt to build a complete RIE evaluation system that reflects the degree of innovation liveliness and the coupling coordination development of innovation habitat, as shown in Table 1. In Innovation Liveliness, in terms of measuring innovation output, we chose the number of patent applications the number of patents granted, and the number of papers published as the main indicators. We utilise it to fully reflect the actual results of innovation. In order to consider innovation input more comprehensively, we used investment in scientific research and the number of scientific research staff as key indicators to objectively assess the actual level of support for innovation activities. In order to more accurately reflect the innovation potential of the region to cultivate and reserve innovative talent, we represented innovation potential through the number of students in general colleges and universities and the number of higher education Institutions. In innovative habitats, to measure infrastructure, we used per capita public finance income, highway mileage, number of hospitals, and the number of teachers in basic education to synthesize the level of infrastructure support in the region. In terms of economic vitality, we selected the import and export value, the entropy of the banking sector location entropy, and retail sales as the key indicators. We utilise it to fully reflect the business vibrancy and economic potential of the region. At the same time, we chose GDP per capita, urban employed population, and the number of mobile phone subscribers to examine the social elemental support system of the region more comprehensively.

**Table 1:** Evaluation Index System of RIE in GBA.

System level	Guideline level	Indicator level
Innovation liveliness	Innovation output	Number of patent applications
		Number of patents granted
		Number of papers published
	Innovation input	Investment in scientific research
		Number of scientific research staff
	Innovation potential	Number of students in general colleges and universities
		Number of higher education Institutions
Innovation habitat	Infrastructure	Per capita public finance income
		Highway mileage
		Number of hospitals
		Number of teachers in basic education
	Economic vitality	Import and export value
		Banking sector location entropy
		Retail sales
	Elemental support	GDP per capita
		Urban employed population
		Number of mobile phone subscribers

### Research methodology

#### CRITIC: entropy weight method

The CRITIC can comprehensively measure the contrast strength and conflict between indicators, but cannot measure the dispersion degree between indicators. While the entropy weight method determines the indicator weights based on the dispersion degree between indicators^28^. The combined use of the CRITIC and the entropy weight method can minimize the loss of information, with the following formula.

First, the indicators are standardized. ${X}_{ij}$ denotes the i-th city and the j-th indicator after standardization. ${X}_{ij}$ represents the i-th city, the raw value of the j-th indicator.

1 $${X}_{ij}=\frac{{x}_{ij}-{x}_{imin}}{{x}_{imax}-{x}_{imin}}.$$

Second, weights are calculated according to the CRITIC. ${w}_{j}^{1}$ is the CRITIC weight of the j-th indicator in the indicator system. $\sigma_{j}$ is the standard deviation of the j-th indicator, and $r_{tj}$ is the correlation coefficient between indicators t and j.

2 $$w_{j}^{1} = \frac{{\sigma_{j} \sum\limits_{t = 1}^{n} {(1 - r_{tj} )} }}{{\sum\limits_{j = 1}^{m} {\left[ {\sigma_{j} \sum\limits_{t = 1}^{n} {(1 - r_{tj} )} } \right]} }}.$$

Third, the entropy value and entropy weight of each indicator are measured. Where n is the number of samples, ${X}_{ij}$ represents the i-th city, the value of the j-th indicator after standardization, ${e}_{j}$ represents the entropy value of the j-th indicator, ${w}_{j}^{2}$ is the entropy weight of the j-th indicator in the indicator system.

3 $$e_{j} = \ln \frac{1}{n}\sum\limits_{i = 1}^{n} {\left( {\frac{{X_{ij} }}{{\sum\limits_{i = 1}^{n} {} }}\ln \left( {\frac{{X_{ij} }}{{\sum\limits_{i = 1}^{n} {X_{ij} } }}} \right)} \right)} ,$$

4 $$w_{j}^{2} = \frac{{1 - e_{j} }}{{\sum\limits_{j = 1}^{m} {(1 - e_{j} )} }}.$$

Fourth, the score of each system is calculated by combining the weights. ${U}_{i}$ is the composite score of the i-th city, ${w}_{j}^{1}$ and ${w}_{j}^{2}$ are the CRITIC weights of the j-th indicator in the indicator system and the entropy weights of the j-th indicator in the indicator system, respectively.

5 $$U_{i} { = }\sum\limits_{i = 1}^{n} {\left( {\frac{{w_{j}^{1} + w_{j}^{2} }}{2}} \right)} X_{ij} .$$

#### Coupling coordination model

Coupled coordination describes the development process of subsystems from disorder to order in evolution. The coupled coordination model can effectively quantify the degree of coordinated development of the system with the following formula^29–31^.

Coupling degree as:

6 $$C = 2\sqrt {\frac{{U_{1} U_{2} }}{{\left( {U_{1} + U_{2} } \right)^{2} }}} ,\;0 \le C \le 1.$$

Composite harmonization index as:

7 $$T = aU_{1} + bU_{2} ,\;a = b = 0.5.$$

Coupling coordination as:

8 $$D{ = }\sqrt {C \times T} ,$$

where ${U}_{i}$ is the value of each subsystem, in most studies, it is assumed that the importance of each subsystem is the same, in this paper. We assume that the weight of a, b for the corresponding two subsystems, respectively, are 0.5, and the coupling degree C, the composite harmonization index T and the coupling coordination degree D take values between 0 and 1, the higher the value the better.

#### Standard deviation ellipse method

The standard deviation ellipse method is a spatial statistical method. It can quantitatively analyze the multidimensional spatial characteristics of an attribute in the region by the changing characteristics of the centre of gravity, declination, area, long semi-axis, and short semi-axis of the ellipse with the following formula^32^:

9 $$X = \sum\limits_{i = 1}^{n} {D_{i} } x_{i} \bigg/\sum\limits_{i = 1}^{n} {D_{i} } ,\;Y = \sum\limits_{i = 1}^{n} {D_{i} y_{i} }_{i} \bigg/\sum\limits_{i = 1}^{n} {D_{i} } ,$$

10 $$\tan \theta = \frac{{\left( {\sum\limits_{i = 1}^{n} {\tilde{x}_{i}^{2} - } \sum\limits_{i = 1}^{n} {\tilde{y}_{i}^{2} } } \right) + \sqrt {\left( {\sum\limits_{i = 1}^{n} {\tilde{x}_{i}^{2} - } \sum\limits_{i = 1}^{n} {\tilde{y}_{i}^{2} } } \right) + 4\left( {\sum\limits_{i = 1}^{n} {\tilde{x}_{i} } \tilde{y}_{i} } \right)^{2} } }}{{2\sum\limits_{i = 1}^{n} {\tilde{x}_{i} } \tilde{y}_{i} }},$$

11 $$\delta_{x} = \sqrt {\sum\limits_{i = 1}^{n} {(\tilde{x}_{i} \cos \theta - \tilde{y}_{i} \sin \theta )}^{2} /n} ,\;\delta_{y} = \sqrt {\sum\limits_{i = 1}^{n} {(\tilde{x}_{i} \sin \theta - \tilde{y}_{i} \cos \theta )}^{2} /n} ,$$

where X and Y are the areal coordinates of the coupling coordination RIE, $x_{i}$ and $y_{i}$ are the geographic coordinates of cities. $\theta$ is the angle formed by rotating clockwise in the due north direction to the long axis of the ellipse. $\tilde{x}_{i}$ and $\tilde{y}_{i}$ are the deviation of the geographic coordinates of each city to the centre of gravity. $\delta_{x}$ and $\delta_{y}$ are respectively the standard deviations along the x-axis and y-axis.

#### Social network analysis method

The social network analysis method is for the relationship data between nodes in the regional space, specific analysis of the individual status, network connection pattern, and overall network structure. Its basic assumption is that the importance of the node depends on the significance of the node with other nodes’ connection^33^.

The spatial connection quantity of coupling coordination is used to describe the interconnectedness of the coupling coordination degree among cities^12^. We refer to the previous research results^12,34^ to construct the spatial association matrix of RIE coupling coordination based on the gravitational force model and measure the connection of coupling coordination between cities. Meanwhile, we break through the limitation of geographical distance without considering the geographical traffic impedance to more scientifically measure the spatial transaction costs between each city^18^. We used Baidu map API to obtain the time cost between nodes to optimize the coupling coordination gravitational model with the following formula.

12 $$R_{ij} = k \times \frac{{D_{i} \times D_{j} }}{{t_{ij}^{2} }}.$$

$R_{ij}$ is the coupling coordination spatial connection strength, $D_{i\left( j \right)}$ is the coupling coordination degree of the city i(j), $t_{ij}$ is the time distance, and the gravitational constant k takes 1.

The point degree centrality of the nodes in the network reflects the status of direct connection with other cities, and the larger the point degree centrality, the stronger the relationship resources occupied by the nodes. The formula is

13 $$C_{ij} { = }\frac{{\sum\limits_{j = 1,i \ne j}^{n} {l_{ij} } }}{n - 1}.$$

$l_{ij}$ is the strength of the inter-city connection and n is the number of nodes.

The eigenvector centrality of a node measures the “quality” of a city’s connection to other cities, that is it depends on the importance of the neighbouring cities. The formula is

14 $$EC\left( {\text{i}} \right) = x_{i} = c\sum\limits_{j = 1}^{n} {a_{ij} x_{j} } .$$

$c$ is the proportionality constant and $x$ is the important measure, denoted $x = \left[ {x_{1} ,x_{2} ,x_{3} ,...,x_{n} } \right]^{T}$.

### Study area and data processing

To explore the coupling coordination mechanism and Spatio-temporal evolution characteristics of RIE under a holistic framework, we take 11 cities in GBA as the spatial scope and 2010–2019 as the temporal scope. The data for the research design was mainly derived from the yearbooks of the cities, the Guangdong Provincial Statistical Yearbook, the Guangdong Social Statistical Yearbook, the China Statistical Yearbook, the Hong Kong Statistical Yearbook, the Macao Statistical Yearbook, as well as the official data of the Hong Kong Government Statistics Office and the Macao Statistics and Census Bureau and the World Bank. It should be noted that the data on papers are obtained from the Web of Science core collection database. The data on patents was obtained from the State Intellectual Property Office. The time cost required to construct the regional innovation ecological network was obtained from the Baidu Map API. Indicators containing monetary amounts were converted to RMB at the current year’s exchange rate.

### Overview of the study area

GBA, located along the southern coast of China (111° 21′–114° 53′ E, 21° 28′–24° 29′ N), consists of the two Special Administrative Regions of Hong Kong and Macao, and the nine Pearl River Delta (PRD) cities of Guangdong Province, including Guangzhou, Shenzhen, Zhuhai, Foshan, Zhongshan, Jiangmen, Huizhou, Dongguan and Zhaoqing, with a total area of 56,098 square kilometers. The region has long been one of the most open and economically vibrant areas in China, with a well-developed industrial system, obvious cluster advantages, and a far-leading economic level. By the end of 2022, the resident population of GBA was 86,290,400, accounting for 5.98% of the country’s total population. In 2022, the region’s GDP totaled more than RMB 13 trillion, accounting for approximately 11% of the country’s total GDP.

## Empirical analysis

### Spatio-temporal evolutionary characteristics of the coupling coordination

#### Time-series evolution of the coupling coordination

**Figure 2:**
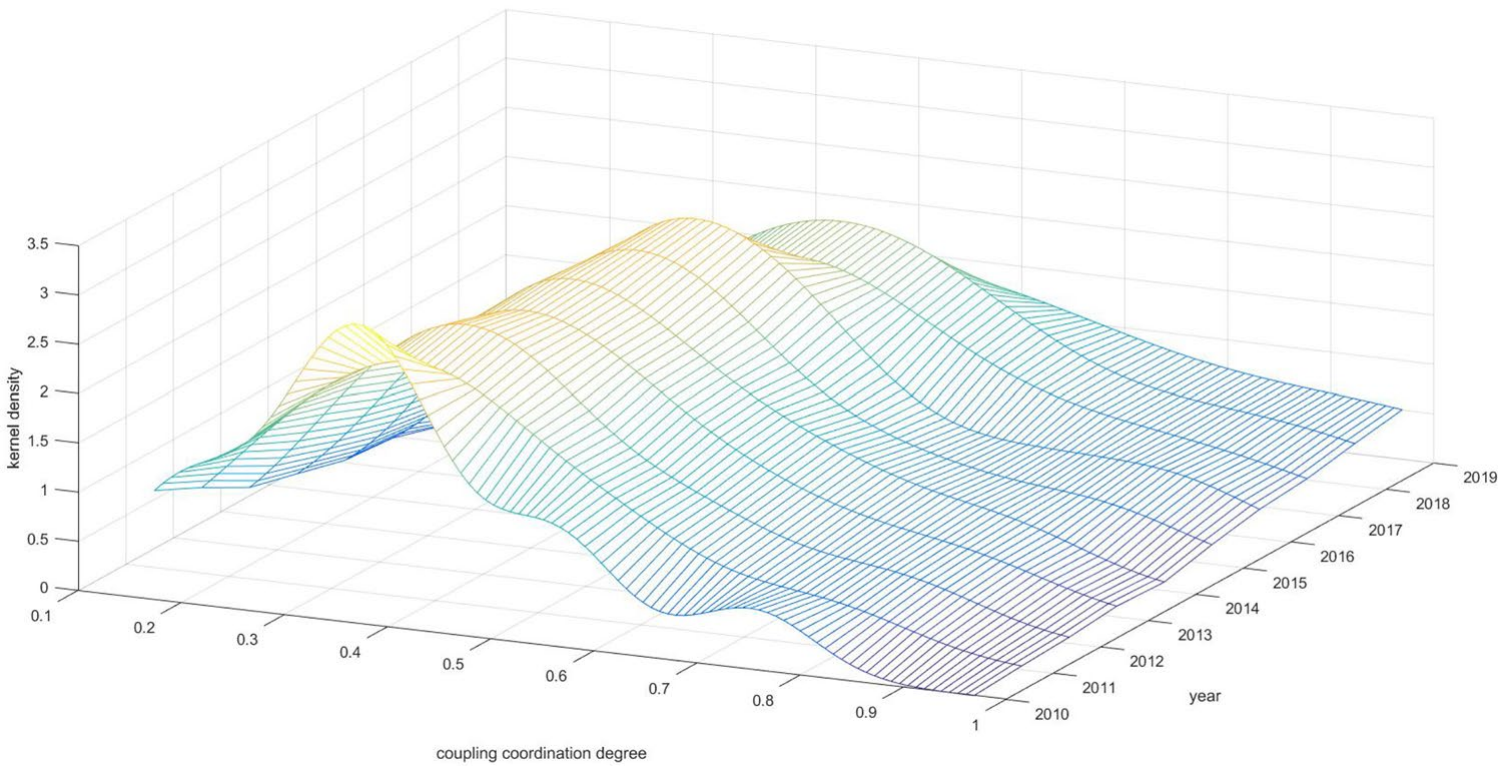
Kernel density estimation of coupling coordination degree.

Kernel density estimation curves are applied to measure the dynamic evolution characteristics of the coupled coordination of innovation activity and innovation habitats in GBA, as shown in Fig. 2. From the shape, the kernel density functions are all single-peak structures, indicating that the coupled and coordinated development of the IE has not shown obvious polarization characteristics. From the distribution position of the curves, the kernel density curves show a right-skewed distribution. The distribution curves for 2010–2019 show an overall rightward trend, indicating that the overall state of coupled coordination is continuously optimized. In terms of wave crests, 2010 was at a low level of harmonization. From 2011 to 2016, the main wave crests showed a trend of rising heights and narrowing widths, with a narrowing of distribution intervals, suggesting a gradual narrowing of the overall gap. It suggests the existence of a “club convergence” within the region, as well as an improvement in regional balance. In contrast, the wave height fluctuation decreased and the width increased in 2017–2019, indicating that the differences in the coupled and coordinated regional development show a fluctuating and widening trend. The trade war between China and the United States broke out in 2019. GBA, as a world-class city cluster with the obvious characteristics of an externally oriented economy, is subjected to a severe economic test. Retail sales and the import and export values were disturbed to a certain extent, especially the weaker economic strength of the city with large populations shows poorer risk resistance. It coupled with the degree of coordination of the horse-tracing effect of “the strong are always stronger, the weak are always weaker”.

#### Centre-of-gravity migration and standard deviation elliptic case

**Figure 3:**
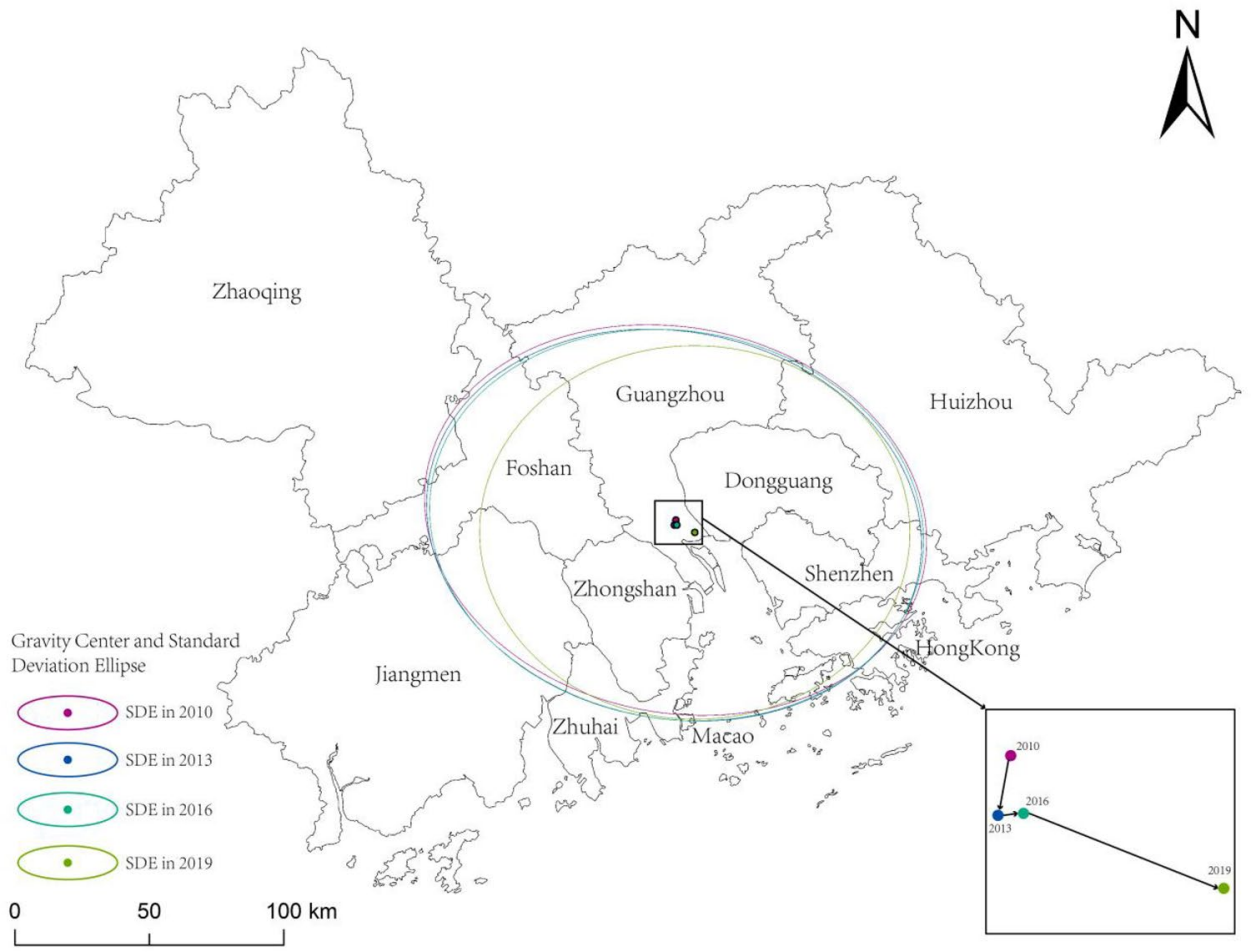
Kernel density estimation of coupling coordintion degree. Illustrated by authors using ArcMap sofware (Esri Inc. (2019). ArcMap 10.8, https://www.esri.com/en-us/arcgis).

The development direction and migration of the coupling coordination are revealed with the help of the centre of gravity model and the standard deviation ellipse method, as shown in Fig. 3. From the migration of the centre of gravity, the centre of gravity moves 1.95 km to the southwest, 0.86 km to the northeast, and 6.84 km to the southeast from 2010 to 2019, respectively. It can be observed that the center of gravity shifted significantly to the southeast in 2016–2019. It also confirms the effectiveness of Shenzhen’s social policy for talent introduction since 2017. In the same year, the governments of Shenzhen and Hong Kong joined hands to create the “Shenzhen-Hong Kong Science and Technology Innovation Cooperation Zone”, which has significantly increased its momentum. The zone and the Hong Kong park built a number of high-end scientific research projects, in a short period of time to form from zero to the development pattern of agglomeration. Meanwhile, it attracts a large number of high-tech talent. Overall, the centre of gravity of coupling coordination shows a trend of moving to the southeast, and the distance between the beginning and the end of the centre of gravity is 7.97 km. It indicates that the development level of coupling coordination in the southeast of GBA is better than other regions, and the advantage of the Shenzhen-Hong Kong cluster is outstanding. The advantages of collaborative innovation are outstanding. In addition, the centre of gravity from 2010 to 2019 is located in the south of Guangzhou. It indicates that Guangzhou is the “innovation brain” of GBA and is a key node in unifying the IE of the cities in the region. From the standard ellipse difference, the main axis shows a northeast-southwest direction. From 2010 to 2019, the turning angle decreased by 15.23, and the standard difference between the main axis, and the secondary axis respectively decreased by 1.36 km and 0.18 km. The ellipse area decreased by 3482.88 km. This indicates that the coupling coordination shows a clustering trend of migration to the southeast, and the spatial coupling coordination strengthens year by year and tends to polarize distribution.

#### Trend surface expression of the coupling coordination

**Figure 4:**
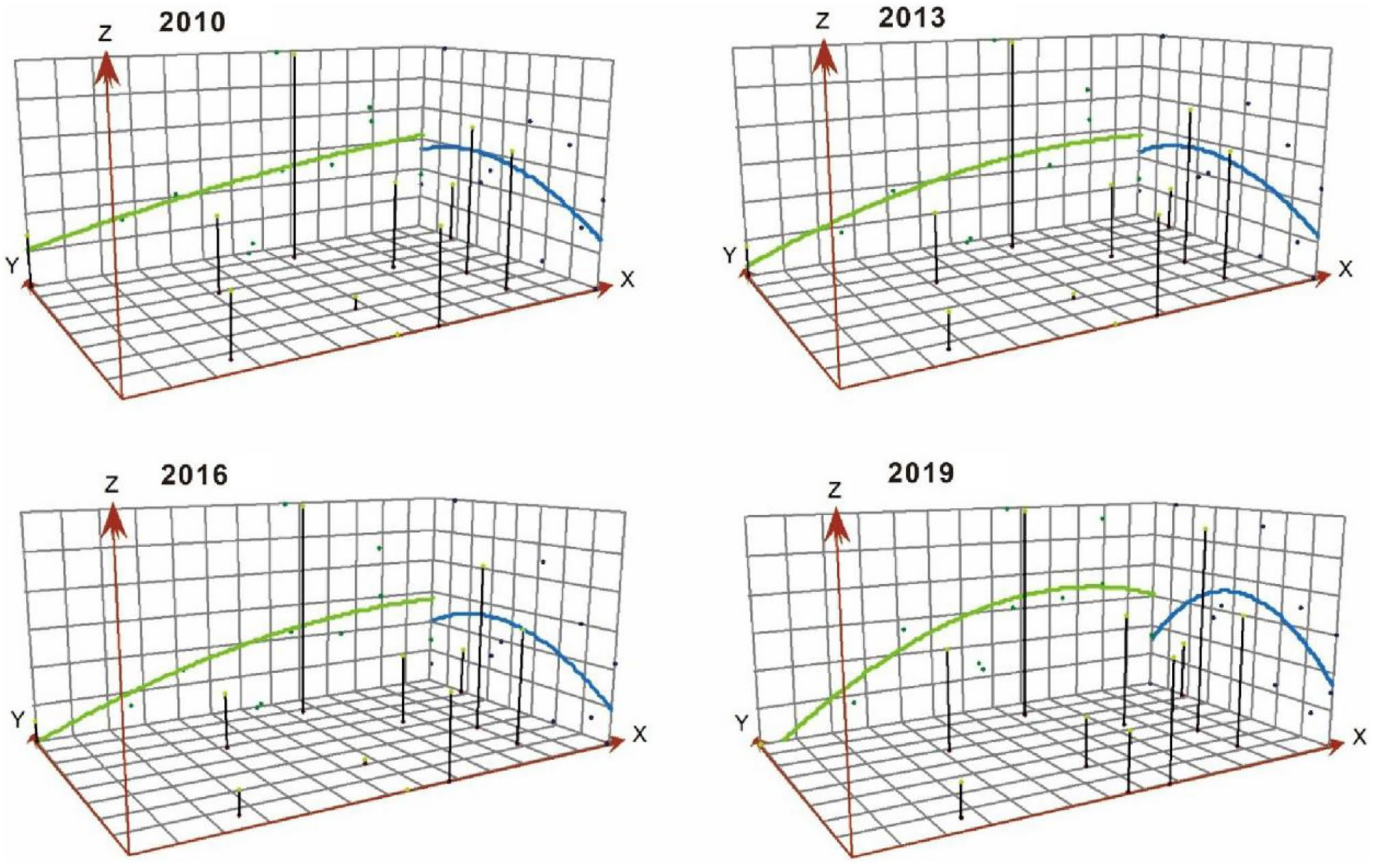
Trend surface analysis of coupling coordination degree. Illustrated by authors using ArcMap sofware (Esri Inc. (2019). ArcMap 10.8, https://www.esri.com/en-us/arcgis).

We use the trend surface analysis tool in ArcGIS to spatially visualize the coupling and coordination through smooth mathematical surfaces, as shown in Fig. 4.

From the characteristics of the curve change, in the north–south direction, it shows a “U” curve of “high in the middle and low on both sides—north is higher than south”. Additionally, the slope and arc are increasing, and the position of the apex is gradually shifting in the middle. Guangzhou is at a high value of coupling coordination and is located in the middle and north. With the improvement of the coupling coordination level of Shenzhen, Hong Kong, and Macao, the difference between north and south is narrowing, and the regional balance is improved to some extent. In the east–west direction, the upward evolution trend of the central region becomes more and more obvious, showing a pattern of change from a smooth curve to a gradually enhanced parabola. The coupling coordination low-value area is concentrated on the west bank of the Pearl River, Zhaoqing, Jiangmen, Zhongshan, and Zhuhai have not yet produced a significant benign coupling interaction, showing a certain disorder regional lock phenomenon.

Whether in the north–south or east–west direction, the curve always maintains a transmutation trend towards the middle. Obviously, the spatial direction of the coupling coordination development of the RIE in GBA is more obvious. The level of coupling coordination development of the cities along the “Guangdong–Shenzhen–Hong Kong–Macao Science and Technology Innovation Corridor” becomes more and more prominent. In addition, it gradually forms an innovation highland that gathers innovation resources and then drives the high-quality development of the RIE. It is an advantageous space for the benign interaction of innovation in GBA.

### Analysis of spatial connections of regional innovation ecological networks

#### Spatial characteristics of the overall network with coupling coordination connection

**Figure 5:**
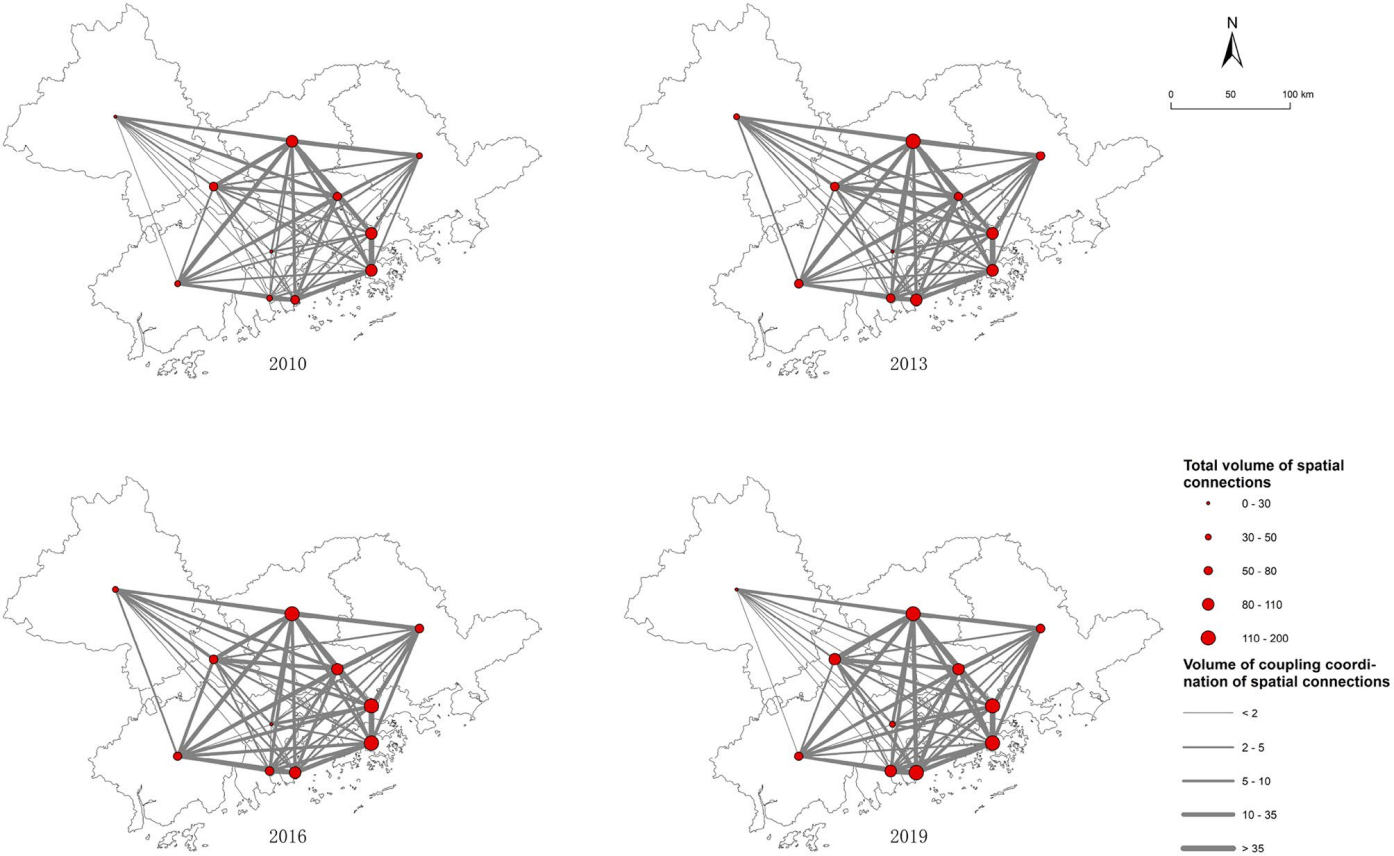
Map of dynamic network structure of spatial connection of GBA. Illustrated by authors using ArcMap sofware (Esri Inc. (2019), ArcMap 10.8, https://www.esri.com/en-us/arcgis).

The above paper describes the evolutionary characteristics of the spatial structure of the coupling coordination between innovation liveliness and innovation habitat. However, it is difficult to reflect the coupling coordination connections and the special status of each node in the RIE. To reveal the “black box” structure within the RIE, we break through the limitation that traditional indicators such as the Moran index, coefficient of variation, and Gini coefficient can only measure the overall degree of association of the RIE. Measuring the spatial connection value of coupling coordination among cities in GBA with the help of the gravity model. Afterward, we constructed the regional innovation ecological network from 2010 to 2019 with ArcGIS to construct the regional innovation ecological network structure from 2010 to 2019, as shown in Fig. 5.

In terms of the overall structure of the network, with the flow of innovation factors in the region, the coupling coordination spatial connections in GBA are getting closer and closer. The total amount of connections among cities increases year by year and gradually forms a polycentric connection development trend. During the dynamic evolution of the innovation ecological network, the city pairs with relatively prominent spatial connection volumes are Shenzhen-Hong Kong, Guangzhou-Foshan, and Zhuhai-Macao. It is not difficult to find that these city pairs have a deep historical cooperation foundation. They are also located in the core area of the central axis and adjacent to each other. It also indicates that there is a significant spatial transaction cost in the innovation ecological network of GBA, that is, there is a spatial frictional diffusion effect of the first law of geography. Cities in close geographical proximity enjoy more convenience and speed in many aspects. It includes the exchange and interaction of talents, absorption and sharing of technology and knowledge, interconnection of capital investment, etc. These locational advantages promote the cooperation and connection of innovation liveliness and innovation habitat coupling coordination development. In 2010, coupling coordination strong ties were established between Shenzhen and Hong Kong. Shenzhen has the leading domestic science and technology innovation industry chain, while Hong Kong has the world’s leading talent base and financial vitality. The two complement each other’s strengths and form a strong innovation synergy through cluster collaboration.

As the “innovation backbone” of GBA, the radiation diffusion effect of the Guangdong–Shenzhen–Hong Kong-Macao Science and Technology Innovation Corridor is becoming more and more obvious. It drives the rapid rise of Zhuhai, Foshan, Dongguan, Huizhou, and other neighbouring cities. The number of stronger connections or even stronger connections in the region is rapidly rising, forming a synergistic development of pole-driven, axis-supported, and radiation-periphery network structures. However, the regional innovation ecological network also shows vulnerability in the face of the external impact of the China-US trade war in 2019. Jiangmen and Zhaoqing, which are geographically disadvantaged in the network, have significantly reduced the level of connections with other cities. It leads to the two cities’ difficulty generating effective synergies with the core cities to jointly resist shocks, and risk marginalization and isolation.

#### Analysis of the centrality of regional innovation ecological network

**Figure 6:**
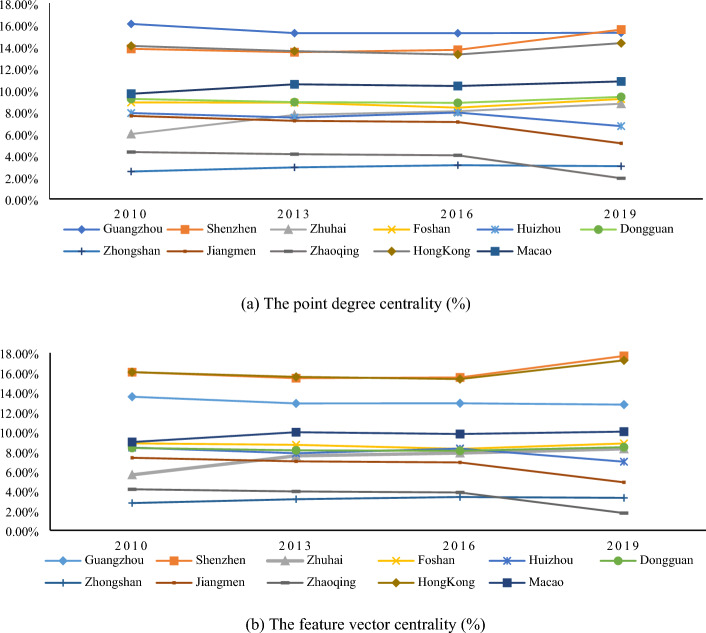
Centric degree of coupling spatial connection between GBA.

We calculate the point degree centrality and feature vector centrality of each city in different years. Aiming at exploring the specific position and role of each node city in the regional innovation ecological network in GBA, as shown in Fig. 6.

From the point of degree centrality, Guangzhou, Shenzhen, and Hong Kong firmly hold the core position and are the three nodes with the strongest control in the network. They release positive radiation to surrounding cities through the diffusion effect, driving the coupling coordination benign development of the whole region. As the “innovation brain” and political centre of the GBA city cluster, Guangzhou has absolute control over the innovation resources and cooperation relations in the network and is the “bridge” to promote cross-regional collaborative innovation cooperation. In recent years, Shenzhen, with the advantages of gathering high-end elements such as financial capital and scientific and technological talents, has grown rapidly in terms of the point degree centrality. Shenzhen’s point degree centrality surpassed Guangzhou for the first time in 2019, becoming a new growth pole of coupling coordination development in the regional innovation ecological network.

In terms of feature vector centrality, the influence of Shenzhen and Hong Kong is significantly higher than that of Guangzhou and has increased significantly in 2019. Shenzhen and Hong Kong occupy the geographical advantage of their locations and generate positive innovation interactions, and the “strong alliance” promotes the enhancement of each other’s coupling coordination development. It also indicates that their development paths of deepening urban innovation cooperation through cluster effects significantly promote each other’s influence in the regional innovation ecological network.

It is not difficult to find that Guangzhou, Shenzhen, and Hong Kong always have strong control over the overall network. While Zhuhai, which was in the synchronous development stage in 2010, has seen a faster rise in the degree of both types of centres and a significant increase in its importance in the network. Zhaoqing, Jiangmen, and Huizhou, which are located on the fringes of GBA, failed to form a benign interactive structure with other cities, and both types of centrality saw a sudden drop in 2019.

#### Core edge structure of regional innovation ecological network

The continuous core–edge model is used by UCINET to measure the number of nuclei of each city node in GBA from 2010 to 2019 respectively. Then we quantitatively analyze the structural characteristics and transmutation patterns of the core, semi-core, and edge areas of the network, and the results are shown in Table 2.

**Table 2:** Core–edge analysis results of regional innovation ecological network in GBA.

Type	2010	2013	2016	2019
Core Zone	Guangzhou Shenzhen Hongkong	Guangzhou Shenzhen Hongkong Macau	Guangzhou Shenzhen Hongkong Macau	Guangzhou Shenzhen Hongkong Macau
Semi-core Zone	Macau Foshan Dongguan Huizhou	Foshan Dongguan Zhuhai Huizhou	Foshan Dongguan Zhuhai Huizhou Jiangmen	Foshan Dongguan Zhuhai Huizhou
Edge Zone	Zhuhai Jiangmen Zhongshan Zhaoqing	Jiangmen Zhongshan Zhaoqing	Zhongshan Zhaoqing	Jiangmen Zhongshan Zhaoqing

It is clear that the “core–edge” spatial structure of the regional innovation ecological network is significant and evolves. Macao and Zhuhai jumped into the core and semi-core zones respectively in 2013, and the network influence of the Zhuhai–Macau cluster began to steadily increase. Jiangmen was among the semi-core areas in 2016 and then fell back to the edge in 2019 due to the external economic situation. Overall, the network structure of multi-core synergy-driven development of Guangzhou, Shenzhen, Hong Kong and Macao is beginning to take shape. Under the influence of the radiation effect of the core poles, semi-core cities develop rapidly and establish a stable connection with the core area. At the same time, the semi-core cities have not yet fully played their role as a connection. They have relatively few cooperative ties with the peripheral cities, risking the solidification of cooperative objects and the rigidity of contacts. The peripheral areas have not yet established a stable leapfrog path, and there is a certain regional lock-in effect.

In 2019, the superimposed impact of the dual factors of domestic economic restructuring policies and international trade frictions between China and the US brought great challenges to GBA. GBA is at a critical stage of the transformation of old and new dynamics. The network structure of the polycentric network pattern has led to a closer synergy between core and semi-core cities. The flat structure has dispersed external risks and strengthened the structural resilience of the core region. However, edge cities show poorer risk tolerance and adaptability. Their innovation ties with other cities become more unstable aftershocks, making it difficult to form a cluster synergy to resist crises together. Therefore, in the face of sudden changes in the external environment, the regional innovation ecological network in GBA exhibits dual characteristics of robustness and vulnerability.

## Results and discussions

Drawing from ecosystem theory and complex network concepts, our study constructs a Regional Innovation Ecosystem (RIE) characterized by the integrated coordination of innovation liveliness and habitat. We investigate the spatio-temporal evolution and network structure characteristics of the RIE in the Guangdong–Hong Kong–Macao Greater Bay area (GBA) from 2010 to 2019. Our methodologies include the CRITIC-entropy weight method, coupling coordination development model, standard deviation ellipse method, and social network analysis. The main conclusions are:

Core–edge analysis from 2013 to 2019 places Hong Kong, Macao, Guangzhou, and Shenzhen at the geographic core, with Guangzhou, Shenzhen, and Hong Kong as core nodes. Macau is still developing into a full core node. Despite GBA’s outlined plan to harness these four cities as the central engines of development, the actualization of this plan remains incomplete, with innovation networks in the GBA showing a polycentric node structure, and Hong Kong as a notable central node. The results of this study are consistent with other studies indicating that the innovation network in GBA exhibits a network structure of polycentric nodes^35^. Notably, unlike previous studies^35,36^, our study found that Hong Kong is at the center.

Spatial and temporal evolution analyses reveal that from 2010 to 2019, the kernel density curve shifted rightward, indicating continuous optimization of regional coupling coordination. However, the China-U.S. trade friction in 2019 led to a decreased peak and increased width of this curve, highlighting a ‘Matthew effect’. The center of gravity for coupling coordination has consistently moved southeast, suggesting better development in this direction, and the area of the ellipse has decreased annually, indicating a polarization trend. The “Guangdong–Shenzhen–Hong Kong–Macao Science and Technology Innovation Corridor” shows increasing prominence as a favorable space for innovation interaction in the GBA.

The spatial connections in GBA are getting closer. The number of stronger connections, or strong connections, is rapidly rising. This forms a network structure of synergistic development driven by poles, supported by axes, and radiating the periphery. The combinations of cities with relatively prominent spatial connections are all located in the core area of the central axis and adjacent to each other. This indicates that there are significant spatial transaction costs in the regional innovation ecological network. However, the 2019 trade war led to reduced connectivity for cities like Jiangmen and Zhaoqing, raising concerns about their marginalization.

In terms of network centrality, Guangzhou, Shenzhen, and Hong Kong stand out as core nodes. Zhuhai, in particular, has seen a significant increase in centrality over the past decade. The ‘core–edge’ structure of the network is evolving, with semi-core cities rapidly developing under the influence of core city radiation effects. The regional innovation ecological network shows both robustness and vulnerability in the face of external shocks.

Our study’s insights into the spatial structure and driving mechanisms of the RIE’s coupling coordination can aid in the development of science and technology collaborative innovation across the GBA. The recommendations based on our findings include.

Lower transaction costs in space and improve system communication. Improving the comprehensive spatial transportation system of the Guangdong–Hong Kong–Macao Greater Bay Area serves several purposes, which facilitates the construction of the “1-h living circle”. It shortens the time and spatial distance between cities, optimizes the precision and efficiency of resource allocation, and promotes the interconnection of innovation factors within the region. Focusing on the construction of an informal network with the government as the core. This promotes the diversified and balanced development of the region through the guidance of information resources, the transmission of cultural values, and the orientation of policies and measures. Strengthening the legal and property rights protection system of cross-region collaborative innovation to weaken the cost of cross-region cooperation and systematic obstacles for innovation subjects.

The innovation model is adapted to local conditions to improve system diversity. According to the coupling coordination development characteristics of cities and their roles in the network, we formulate development strategies according to local conditions, avoid the phenomenon of homogenization of industrial layout, and deepen the advantages of dislocation development of regional functions. Strengthen the heterogeneous connection between the core and edge cities, and provide targeted assistance in talents, capital, and projects. This strengthens the innovation vitality and structural toughness of the overall network through complementary division of labour. In order to break through the dysfunctional regional lock-in effect, and improve the comprehensive risk-resistance capability of edge cities. To realize the coupling coordination development of the innovation ecological network in GBA, efforts are required at both the city level and the collaborative network level. To realize the coupling coordination development of the innovation ecological network in GBA, efforts are required at both the city level and the collaborative network level. A synergistic and cooperative network should be formed between government departments, industry associations, enterprises, and universities. Leveraging the role of government innovation policy guidance^37^. So the RIE in GBA will show innovation subjects’ multi-level connection, mutual coupling innovation factors, and the integrated development pattern of multi-centre innovation of overall regional collaborative innovation.

Broaden the innovation food chain and make the system more transparent. GBA’s strategic goal is to establish itself as a hub for international science and technology innovation with a global impact. It necessitates that it looks beyond the coupling connections within the RIE and instead looks further into the national IE and even the global IE. Accordingly, it is necessary to increase the IE’s openness in the GBA, and weaken institutional geo-border barriers. Meanwhile, improving the system’s connections internally and externally, expanding cross-provincial and even transnational collaborative innovation cooperation in a variety of fields, industries, and dimensions, and lengthening the innovation food chain at both the upstream and downstream ends. To create a new development pattern in GBA with a high level of innovation, it is also necessary to direct all types of innovative species in the RIE to establish a collaborative and open vision of innovation. This can be done by developing overseas cooperation channels, setting up innovation platforms, and formulating incentive policies.

This paper utilizes the coupling coordination degree model, standard deviation ellipse method, and social network analysis method to analyze the spatial pattern characteristics of RIE coupling coordination and network development posture more comprehensively.

Although the evaluation index system of GBA’s IE constructed in this paper has been improved compared with the previous research. Due to the inconsistency of the statistical caliber of Guangdong, Hong Kong, and Macao, and the difficulty of obtaining the data, it is still difficult for the existing index system to fully reflect the reality of the effectiveness of the innovation vitality and innovation habitats. In future research, the authors intend to utilize Python tools to crawl open-source data with city information to supplement the evaluation index system.

There are relatively few empirical studies on regional innovation eco-logical networks in academia. This paper refers to the construction method of coupled and coordinated spatial correlation networks of the predecessors. Then we choose the gravity model to describe the spatial structural characteristics of the innovation ecological network in GBA. However, the gravitational model suffers from the disadvantages of a lack of quantitative basis for the assumptions and a lack of realistic basis for the parameter settings. So it is no longer adaptable to describe the network structure with complex spatial characteristics. In future research, the authors intend to use the radiation model, weighted opportunity model, or Spatial econometric model^35^, Term Frequency-Inverse Document Frequency (TF-IDF)^36^, Latent Dirichlet Allocation (LDA)^38^, etc. trying to simulate the spatial connection and connectivity strength of regional innovation ecological networks.

In terms of the scope of the study, the research on RIE in this paper is limited to a single city cluster in GBA, and it does not extend the study to regions such as Beijing–Tianjin–Hebei and the Yangtze River Delta, or amplify it to the national or global scale. A more complete study should include three different scales: global, national, and local, and analysis from different research units will lead to more comprehensive conclusions.

## Data Availability

(1) The data in this study are collected in National Bureau of Statistics http://www.stats.gov.cn/ (accessed on 12 February 2022); (2) Statistics bureau of Guangdong province http://stats.gd.gov.cn/ (accessed on 12 February 2022); (3) Census and Statistics Department of the Government of the Hong Kong Special Administrative Region https://www.censtatd.gov.hk/sc/ (accessed on 12 February 2022); (4) Statistics and Population Census Service of the Macao Special Administrative Region Government https://www.dsec.gov.mo/zh-MO/ (accessed on 12 February 2022); (5) The World Bank https://www.worldbank.org/en/home (accessed on 12 February 2022); (6) Web of Science https://www.webofscience.com (accessed on 12 February 2022); (7) China National Intellectual Property Administration https://www.cnipa.gov.cn/ (accessed on 12 February 2022).
